# Rotating magnetic field ameliorates experimental autoimmune encephalomyelitis by promoting T cell peripheral accumulation and regulating the balance of Treg and Th1/Th17

**DOI:** 10.18632/aging.103018

**Published:** 2020-04-07

**Authors:** Tianying Zhan, Xiaomei Wang, Zijun Ouyang, Youli Yao, Jiangyao Xu, Shikang Liu, Kan Liu, Qiyu Deng, Yushu Wang, Yingying Zhao

**Affiliations:** 1 International Cancer Center, Department of Physiology, School of Medical Science, Shenzhen University, Shenzhen, Guangdong, China

**Keywords:** rotating magnetic field, EAE, inflammation, chemokines, Treg cell

## Abstract

Multiple sclerosis (MS) is an autoimmune disease characterized by T cell infiltration and demyelination of the central nervous system (CNS). Experimental autoimmune encephalomyelitis (EAE) is a classical preclinical animal model of MS. In this study, we found that rotating magnetic field (RMF) treatment exerts potential preventive effects on the discovery of EAE, including reducing the severity of the disease and delaying the onset of the disease. The results indicated that RMF (0.2 T, 4 Hz) treatment increases the accumulation of CD4^+^ cells in the spleen and lymph nodes by downregulating the expression of CCL-2, CCL-3 and CCL-5, but has no significant effect on myelin oligodendrocyte glycoprotein (MOG) specific T cell responses. Simultaneously, RMF treatment adjusted the imbalance between regulatory T (Treg) cell and T helper 1 (Th1) cells or T helper 17 (Th17) cells by increasing the proportion of Treg cells and inhibiting the ratio of Th1 and Th17 cell subsets. These findings suggest that exposure to RMF may improve EAE disease by promoting CD4+ cell accumulation into peripheral lymphoid tissue, improving the imbalance between Treg and Th1/Th17 cells. Therefore, as a mild physical therapy approach, RMF, is likely to be a potential way to alter the development of EAE.

## INTRODUCTION

Multiple sclerosis (MS) is illustrated by inflammatory infiltration as well as demyelination in the central nervous system (CNS) causing physical disability [1]. MS has the highest incidence among people with the most productive work and childbearing age [2]. Immunomodulators are being currently used in clinical trials to research on treatment measures for MS, but these drugs are often accompanied by more adverse reactions including headache, liver enzyme abnormalities, viral infections, diarrhea and hair loss. [3]. Although there are many FDA-approved drugs available for MS, there is no cure for MS. The EAE model induces the formation of susceptible mice by myelin oligodendrocyte glycoprotein (MOG) [4]. EAE has many histological characteristics of MS, including active demyelination, oligodendrocytes and axonal loss that may be activated by myelin-specific T cells [5].

Differentiation of CD4+ T cell into Th1, Th17 and other subsets are critical events for the evolution of the disease, and the process of Th cell differentiation is tightly regulated by multiple cytokines and regulatory T cells (Treg). Early studies have shown that interferon (IFN)-γ production of CD4 + Th1 type (Th1) cells is the main effector cell type in the pathogenesis of EAE [6]. Meanwhile, CD4^+^ Th17 cells express various cytokines, including interleukin (IL)-17A, IL-17F, IL-21 and IL-22, which have been shown to play a decisive role in EAE [7]. These immune cell trafficking processes are part of CNS immunity, coordinated by chemokines, which are small chemotactic peptides. Chemokines have a pro-inflammatory capacity that induces extravasation of leukocytes, which promotes migration and penetration of T cells [8]. Chemokines can further impact antigen presentation, cytokine production, effector and memory T cell differentiation, and regulatory T cell (Treg) function [9]. Treg cells sustain immune self-tolerance and avoid various autoimmune disorders [10]. The dysregulation of Foxp3^+^ Tregs function causes unrestrained immune stimulation as well as autoimmunity [11]. In addition, Tregs have played key functions in the treatment of MS as well as EAE because of their capability to preserve peripheral immune tolerance [12]. In general, Tregs inhibit the creation of the pro-inflammatory cytokines by contact-dependent inhibition of Th cells that have been recognized to generate IL-6, IL-17 and IFN-γ [13].

An increasing number of basic research studies is using magnetic fields (MF) as a method of treatment. Magnetic therapy offers a non-invasive, secure as well as easy way to straightforwardly treat injury, pain and inflammation, as well as other kinds of disorders [14]. Previous study has shown that numerous features of endogenous control of inflammation as well as healing exhibit changes in function when exposed to MF [15]. In the current study, we used a rotating magnetic field (RMF) to study the effect of RMF on EAE. RMF device was prototyped by Shenzhen University (China). The biological response of the magnetic field has a relatively pronounced window effects, amplitude, frequency and exposure duration determine greatly whether a bioeffect will occur [14, 16]. Based on our previous studies [17–19], we determined the RMF exposure: 2 hours per day in a RMF with intensity at 0.2 T and rotating frequency at 4 Hz. Our results indicated that RMF could alleviate the progression of EAE, and reduce the inflammatory cell infiltration as well as demyelination of the spinal cord. Our observations indicate that RMF may provide an alternative medication for MS patients.

## RESULTS

### RMF exposure averts the advancement of EAE in mice

**Figure 1:**
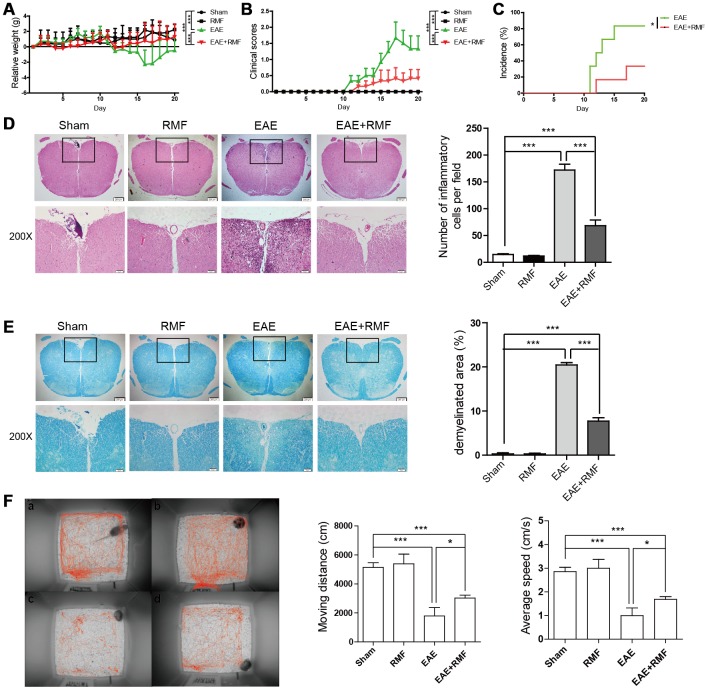
**Protective impact of RMF-exposure on the advancement of EAE in mice.** EAE mice and C57BL/6 mice were treated daily with RMF or non-RMF starting from day 0 following vaccination. Animals were observed for the clinical indications as well as disease advancement of EAE, comprising (**A**) body relative weight, (**B**) clinical scores and (**C**) the percentage of EAE incidence. (**D**) Spinal cord sections of the treated mice on day 20 following vaccination by H&E, Scale bar, 200 μm and 50 μm. The graph shows the number of inflammatory cells in per low magnification field (calculated with animal number: control, n = 3; RMF, n = 3; EAE, n = 6; EAE+RMF, n = 6). (**E**) LFB of Spinal cord sections in both groups on day 20. Scale bar, 200 μm and 50 μm. The graph shows the percentages of demyelinated area in sections (control, n = 3; RMF, n = 3; EAE, n = 6; EAE+RMF, n = 6). On day 20, (**F**) the behavioral trajectory of mice analyzed by rhythm cage: (**A**) Control, (**B**) RMF, (**C**) EAE, (**D**) EAE+RMF. Subsequently, we measured moving distance and average speed of mice within 30 min. Data are shown as the mean ± SEM of three independent experiments. *P <0.05, ***P <0.001.

Active EAE was stimulated in mice as mentioned in the method section. EAE mice and C57BL/6 mice were treated daily with RMF or non-RMF starting from day 0 after immunization. We found that no significant differences in the results of C57BL/6 mice exposed to RMF compared to normal C57BL/6 mice. The results of daily body weight measurements showed that RMF treatment could alleviate the weight loss of EAE mice (Figure 1A). The clinical score for EAE mice were assessed daily, and we observed that RMF treatment could delay the onset and reduce the severity of the disease (Figure 1B). The resulting incidence shows that RMF treatment reduces the incidence of immunized mice by about 40% (Figure 1C). Spinal cords were acquired from EAE mice on day 20 after MOG immunization, which is the time point at which the peak of clinical symptoms of the EAE group [20]. Consistent with the reduction in clinical symptoms of the disease, RMF-treated mice showed markedly diminished inflammation and demyelination in the affected spinal cord, compared with EAE mice (Figure 1D, 1E, P <0.001). Meanwhile, when measuring the behavioral trajectory, we found that the moving distance and velocity of EAE mice after RMF treatment increased, and there was a statistical difference for EAE mice. (Figure 1F).

### RMF mainly affects the immune system of EAE mice

**Figure 2:**
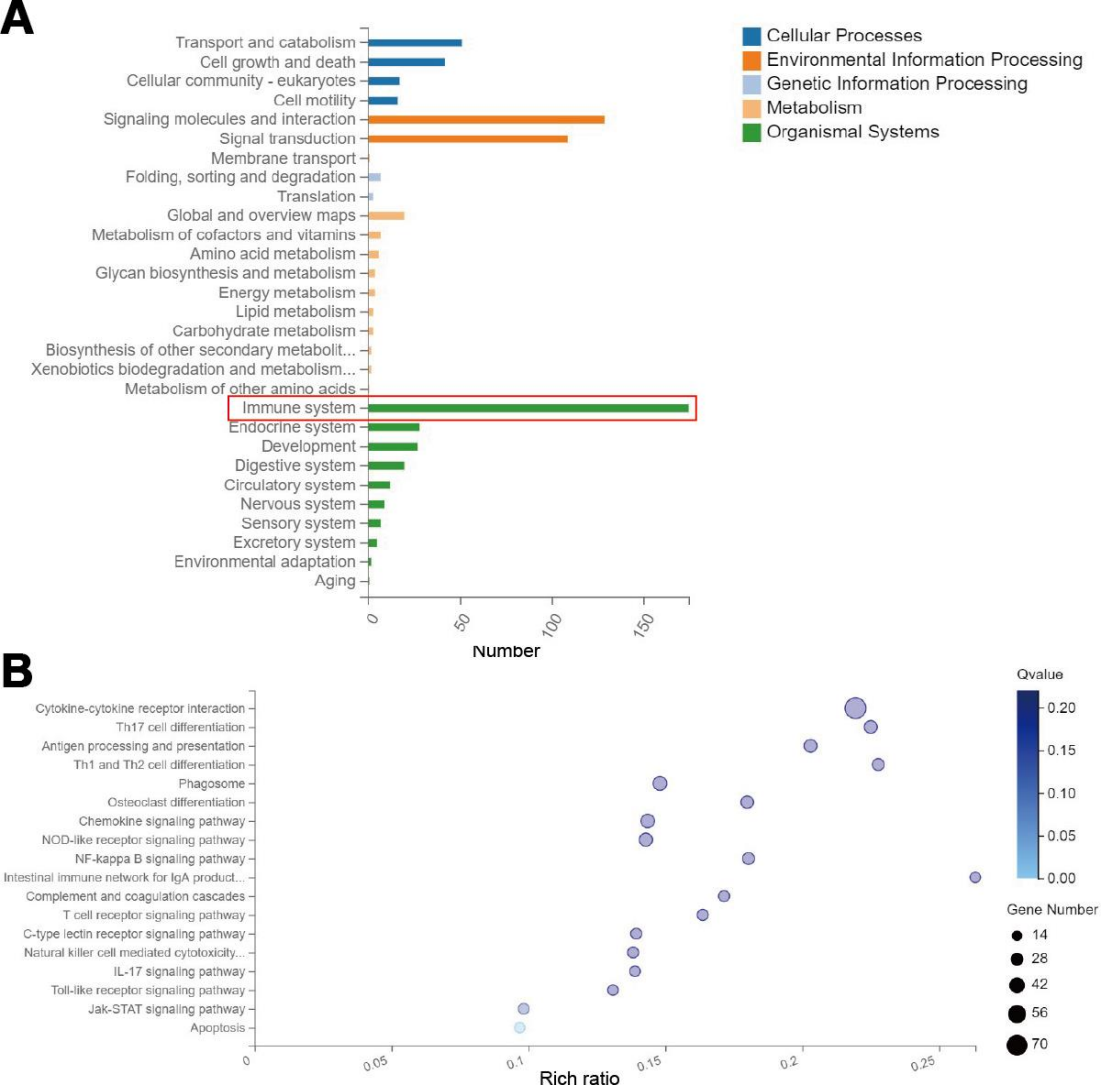
**RMF exposure primarily affects genetic changes in the immune system. Spinal cord was isolated from mice for whole transcriptome sequencing analysis at day 20 after immunization.** (**A**) Classification map of differential gene KEGG pathway. (**B**) Differential gene KEGG pathway enrichment map.

In order to investigate the mechanism by which RMF averts the advancement of EAE in mice, we performed whole-transcriptomics sequencing of the spinal cord. By classifying the differential gene KEGG pathway, we found that the altered genes were mainly concentrated in the immune system (Figure 2A). Then, through the KEGG pathway enrichment analysis, the results showed that the differentially expressed genes were mainly found in following pathways: cytokine-cytokine receptor interaction, Th17 cell differentiation, Antigen processing and presentation, and Th1 and Th2 cell differentiation (Figure 2B). We focused our attention on the immune system for the subsequent analyses that follow.

### RMF has little impact on T cell response

**Figure 3:**
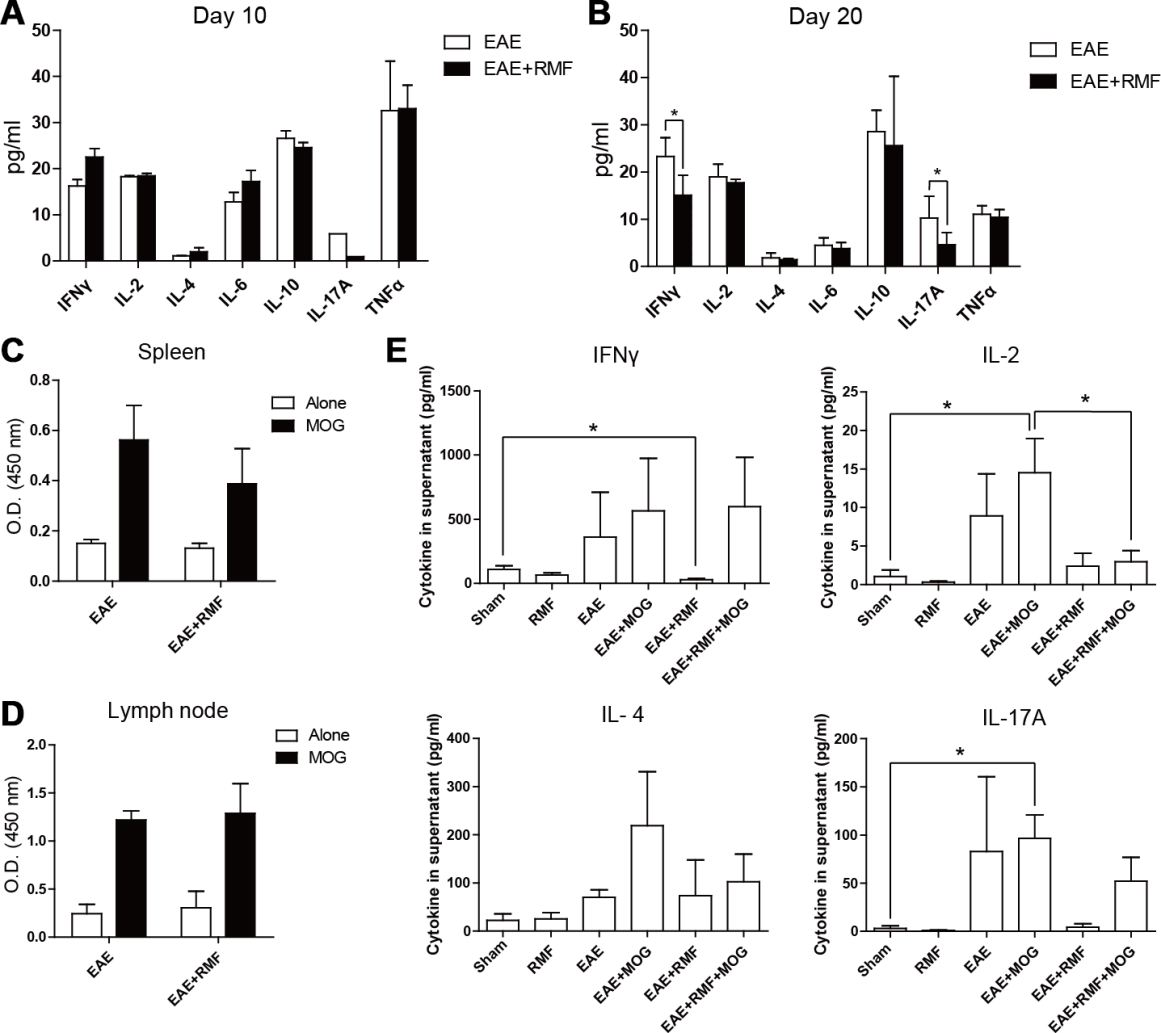
**Minor impacts of RMF on generation of Th1/Th2/Th17 cytokines as well as T cell propagation.** Cytokines in serum from the treated EAE mice were detected at (**A**) day 10 and (**B**) day 20 after immunization. (**C**) Spleen cells and (**D**) lymph node cells derived from the treated EAE mice at day 10 following vaccination were re-activated with 10 μg/mL of MOG35–55 peptide. (**C**, **D**) Propagation was detected after 72 h of incubation. (**E**) IFN-γ, IL-2, IL-4 as well as IL-17A were detected following 48 h of incubation. Data are shown as the mean ± SEM of three independent experiments. *P <0.05.

In order to further validate the results of transcriptome sequencing, we first examined the effect of RMF on the generation of cytokines and T cell propagation. Firstly, the effects of RMF on T-mediated inflammatory response *in vivo* were analyzed. On day 10 post immunization, RMF treatment did not have a large effect on serum levels of cytokines (Figure 3A). On day 20 following vaccination, RMF treatment caused a decrease in cytokine serological levels; IL-17A as well as IFN-γ were most pronounced, and were statistically different compared to the EAE group (Figure 3B). Further, spleen cells and lymph node cells acquired from the EAE mice at day 10 were re-activated with 10 μg/mL of MOG_35–55_ peptide. The two groups showed the same proliferative activity in the stimulation of MOG_35–55_ peptide (Figure 3C, 3D). Subsequently, we examined the cytokines of the spleen cell supernatant and found that the magnetic field treatment reduced the IL-2 level in the co-culture of MOG_35–55_ peptide (Figure 3E). But the other three cytokines did not show statistical differences (Figure 3E).

### RMF treatment stimulates the accumulation of lymphocytes within spleen as well as lymph nodes

**Figure 4:**
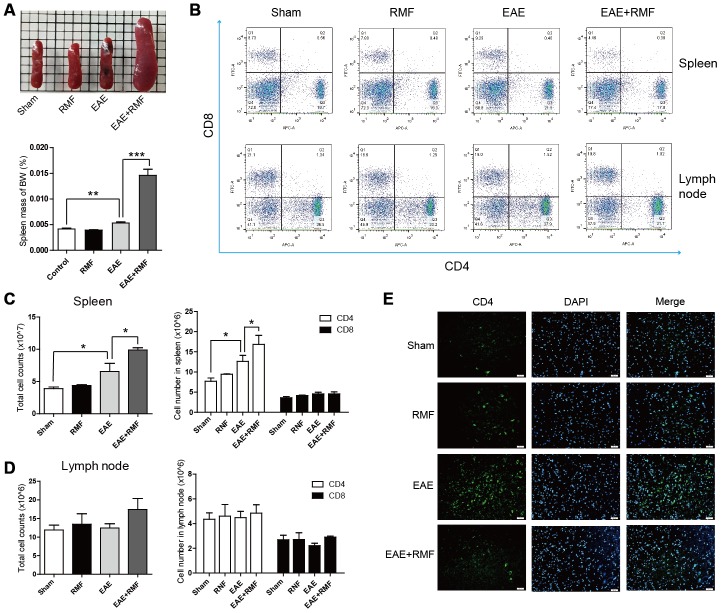
**Impact of RMF on lymphocyte homing to peripheral lymphoid tissues.** (**A**) Demonstrative pictures of the spleen and the mass of the spleen was weighed and the mass of body weight (BW) was calculated. (**B**) Proportions of CD4^+^ as well as CD8^+^ T lymphocytes in the spleen and lymph nodes were determined by flow cytometry. The overall cell numbers as well as CD4^+^ and CD8^+^ cell numbers in the (**C**) spleens and (**D**) lymph nodes. (**E**) Spinal cords from the treated mice at day 20 following vaccination were stained with an antibody specific for CD4. Scale bar, 50 μm. Data are shown as the mean ± SEM of three independent experiments. *P <0.05, **P <0.01, ***P <0.001.

RMF-treated EAE mice were observed having inflamed peripheral lymphoid tissues, for example spleen (Figure 4A). And the spleen mass of body weight (BW) indicated that the spleen of the EAE mice treated with RMF increased significantly (Figure 4A). Therefore, we speculated that RMF could inhibit the transportation of T cells. The proportion of CD4 ^+^ and CD8 ^+^ T lymphocytes in the spleen as well as lymph nodes from RMF treated EAE mice was lower, compared with EAE mice (Figure 4B). The absolute cell numbers increased after RMF treatment, comprising the number of entire spleen as well as draining lymph node cells and their CD4^+^ and CD8^+^ T cell subsets (Figure 4C, 4D). At the same time, the number of CD4^+^ T cells significantly reduced in the RMF-treated EAE group, compared with the EAE group, as a results of specific antibody CD4 localization to the spinal cord (Figure 4E).

**Figure 5:**
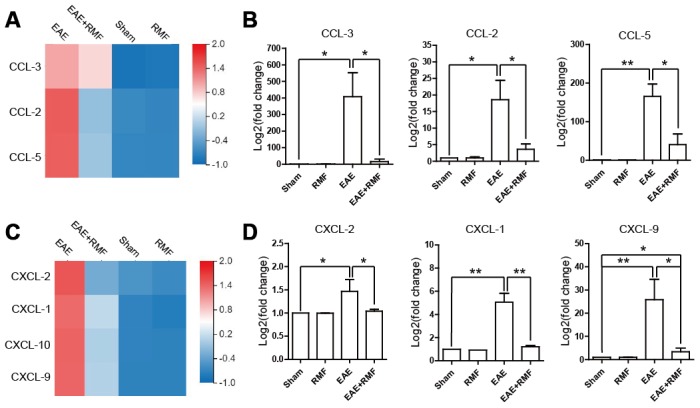
**Effect of RMF on chemokines.** (**A**) Heatmap of CCL-2, CCL-3 and CCL-5 expression difference in the spinal cord. (**B**) The manifestation of CCL-2, CCL-3 and CCL-5 mRNA in spinal cord was measured by qPCR. (**C**) Heatmap of CCL-2, CCL-3 and CCL-5 expression difference in the spinal cord. (**D**) The manifestation of CXCL-1, CXCL-2, CXCL-9 and CXCL-10 mRNA in spinal cord was measured by qPCR. Data are shown as the mean ± SEM. of three independent experiments. *P <0.05, **P <0.01.

Furthermore, we investigated the reason why lymphocytes did not migrate into the spinal cord of magnetically treated EAE mice. Chemokines, such as C-C motif chemokine 2 (CCL-2), C-C motif chemokine 3 (CCL-3) and C-C motif chemokine 5 (CCL-5), have been known to play important roles in peripheral immune adhesion, chemotaxis and migration. We studied the expression of these factors to explain the effect of RMF on peripheral lymphocyte migration. The expression of mRNA in CCL-2, CCL-3 and CCL-5 of EAE mice in spinal cord showed a downward trend after RMF exposure, and the results were statistically significant (Figure 5B). The results were consistent with the sequencing results (Figure 5A). Additionally, both RNA-sequencing and quantitative real-time PCR results showed that the expression levels of IL-17 targeting chemokines, including CXC motif chemokine ligand 1 (CXCL-1) and CXC motif chemokine ligand 2 (CXCL-2) [20], were significantly reduced in RMF treated spinal cord (Figure 5C, 5D). Similar downregulation was also observed for IFN-γ targeting chemokines, such as CXC motif chemokine ligand 9 (CXCL-9) and CXC motif chemokine ligand 10 (CXCL-10) [20] in the RMF treated spinal cord (Figure 5C, 5D). These results indicate that RMF promoted peripheral accumulation in the spleen and lymph node, while the expression of related chemokines in the spinal cord also decreased.

### RMF-treatment changed peripheral CD4^+^ T cell subsets

**Figure 6:**
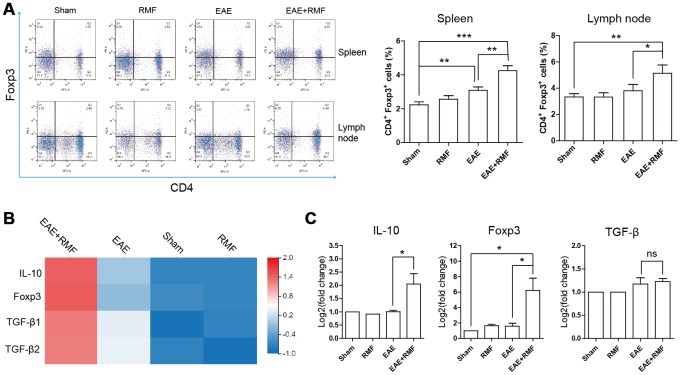
**Impact of RMF on Treg cells.** (**A**) Proportions of Treg cells in the spleen as well as lymph nodes were determined. (**B**) Heat map of TGF-β, Foxp3 and IL-10 expression difference in the spinal cord. (**C**) The manifestation of TGF-β, Foxp3 and IL-10 mRNA in spinal cord was measured by qPCR. Data are shown as the mean ± SEM of three independent experiments. *P <0.05, **P <0.01.

**Figure 7:**
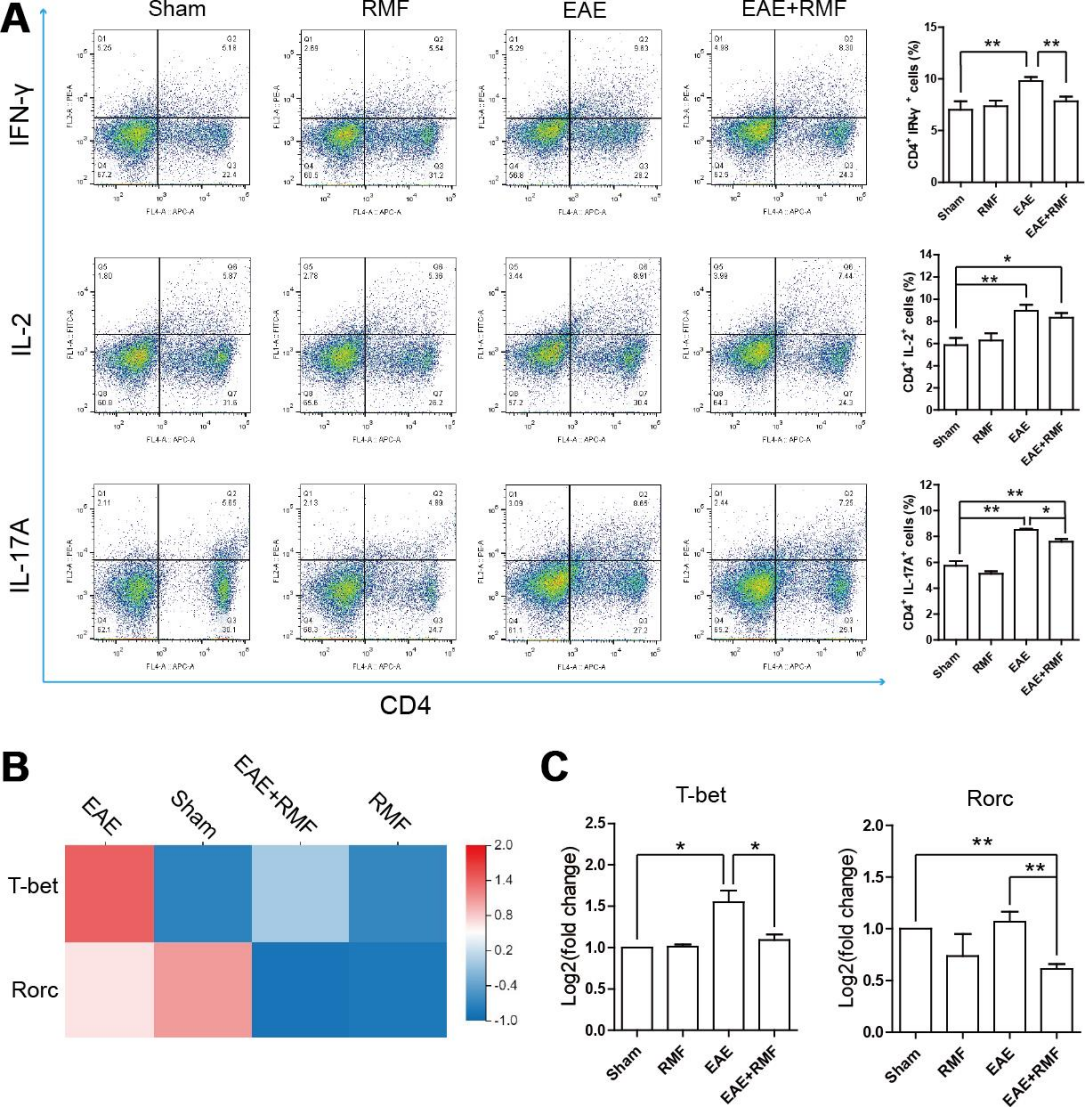
**Impact of RMF on peripheral CD4^+^ T cell subsets.** (**A**) The percentages of CD4^+^IFN-γ^+^, CD4^+^IL-2^+^ and CD4^+^IL-17^+^ cells in the lymph node were detected. (**B**) Heatmap of T-bet and Rorc expression difference in the spinal cord. (**C**) The manifestation of T-bet and Rorc mRNA in spinal cord was measured by qPCR. Data are shown as the mean ± SEM of three independent experiments. *P <0.05, **P <0.01.

The outcomes observed in the spinal cord indicated that there were virtually no CD4^+^ T cells in the spinal cord of EAE mice treated with RMF-treatment. This result suggests that RMF involvement may prevent the development of the spinal cord inflammation microenvironment through direct peripheral immune regulation. Tregs perform a crucial function in preserving peripheral immune tolerance, and Tregs act by inhibiting the effector CD4 + T cell subsets that activate autoimmune responses [21]. At day 20, the spleen and lymph node cells were acquired from mice for FC. The quantity of Treg cells in the spleen and lymph node cells of EAE mice were found to have decreased, after RMF treatment (Figure 6A). Forkhead box P3 (Foxp3) is an indicator for Tregs [22]. Treg cells inhibit Th cells by producing IL-10 and TGF-β [21]. Quantitative real-time PCR and sequencing results showed that RMF treatment up-regulated the level of IL-10, TGF-β and Foxp3 mRNA (Figure 6B, 6C). Then, the distribution of CD4^+^ cell subsets of lymph node cells was examined by flow cytometry (FC), and the outcomes showed that the proportion of CD4^+^IFN-γ^+^, CD4^+^IL-2^+^ and CD4^+^IL-17^+^ cells in EAE mice decreased after RMF treatment (Figure 7A). Tbx21 (T-bet) is a major controller of Th1 cell demarcation [23]. The retinoid-related orphan receptor r-c (RORC) is a major transcription factor has been shown to regulate the differentiation of (Th17) cells and form a synergy with other transcription factors to stimulate IL-17 manifestation [24]. As expected, RMF treatment downregulated the levels of Rorc and T-bet mRNA in EAE mice (Figure 7B, 7C).

**Figure 8:**
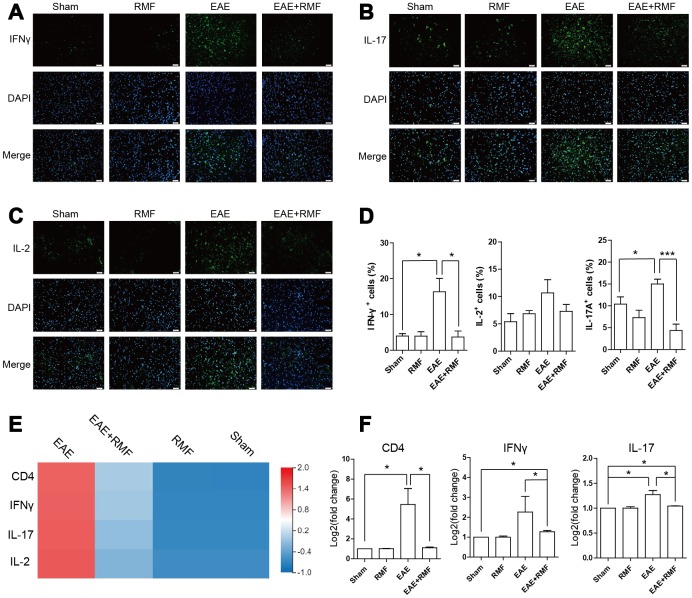
**Effect of RMF on spinal cord.** Spinal cords from the treated mice at day 20 after immunization were stained with an antibody specific for (**A**) IFN-γ, (**B**) IL-2, and (**C**) IL-17. Scale bar, 50 μm. (**D**) The diagram presents the proportion of positive cells in EAE lesions or corresponding areas (control, n = 3; RMF, n = 3; EAE, n = 6; EAE+RMF, n = 6). (**E**) Heatmap of IFN-γ and IL-17 expression difference in the spinal cord. (**F**) The expression level of CD4, IFN-γ and IL-17 mRNA in spinal cord was measured by qPCR. Data are shown as the mean ± SEM of three independent experiments. *P <0.05, **P <0.01, ***P <0.001.

Furthermore, in order to determine whether changes in the subpopulation of infiltrated CD4^+^ T cells in CNS were consistent with the results of peripheral lymphoid tissues, we analyzed spinal cord with IFN-γ, IL-2 and IL-17 specific antibodies. It was found that the expressions of IFN-γ, IL-2 and IL-17 in the EAE spinal cord decreased significantly after RMF exposure (Figure 8A–8C). The similar results can be obtained by calculating the percentage of positive cells in each field of view (Figure 8D). Quantitative real-time PCR and sequencing results showed that RMF treatment down-regulated the expression levels of IFN-γ and IL-17 in the spinal cord of EAE mice (Figure 8E, 8F). These results were found to be consistent with the results of peripheral lymphoid tissue.

## DISCUSSION

Hypotheses for the pathogenesis of EAE have been proposed [25–27]. After antigen-specific T cells are elicited in peripheral tissues, they enter the subarachnoid space and encounter antigens presented by macrophages and are re-stimulated. After the amplification of proliferation, cytokines are up-regulated and released into CNS tissues. Substantial vasculature is subsequently activated by cytokines, such as IFN-γ and IL-17; and perivascular inflammatory cell infiltration accumulates, resulting in an explosive inflammatory cascade and motor weakness related to the beginning of EAE. In this study, we established that RMF exposure has a preventive effect on the advancement of EAE in mice. RMF treatment suggestively reduces the severity of EAE. In order to clarify the efficiency of RMF to alleviate EAE. firstly, we evaluated the effects of RMF exposure on T cell stimulation. *In vivo* experiments revealed that RMF treatment did not influence MOG-specific T cell responses, comprising T cell propagation and cytokine generation. However, on day 20 after immunization, IFN-γ and IL-17A levels in the serum of EAE mice were significantly reduced by the exposure of RMF. The results suggested that RMF exposure could inhibit Th1/Th17 cell subsets in peripheral lymphoid tissues.

Interestingly, we observed significant swelling of the mice spleen during the experiment. Studies have shown that controlling lymphocyte migration to uninfected CNS can regarded as an effective therapeutic approach for the initial stage of EAE [26]. For example, α4 integrin inhabits the transport of lymphocytes into the CNS, allowing for CNS autoimmune inflammation to be resolved by reducing the quantity of lymphocytes in the CNS [28]. Fingolimod (FTY720) prevents lymphocytes from escaping from the lymph nodes along with reduces the penetration of inflammatory mediators into the CNS [29, 30]. In the current study, RMF treatment caused the accumulation of CD4^+^ and CD8^+^ cells in lymph nodes as well as spleen. We observed that RMF exposure diminished the penetration of CD4^+^ cells into the spinal cord at the peak of EAE. We speculated that RMF enhances the homing reaction of lymphocytes, causing lymphocytes to accumulate in peripheral lymphoid tissues, and making it impossible for CD4^+^ cells to migrate into the spinal cord, thereby alleviating inflammation of EAE. Based on sequencing results, we found that RMF exposure significantly reduced the expression of chemokines from sequencing results, and that simultaneous detection using real-time quantitative PCR also yielded the same results in the spinal cord and spleen. Our results showed that RMF could strongly down-regulate chemokines such as CCL-2, CCL-3 and CCL-5 in peripheral immune cells, thereby preventing lymphocytes from migrating to the spinal cord to reduce the extent of inflammation. In EAE mice, expression of CCL-2 mRNA in the brain and spinal cord is up-regulated and may be able to mediate the onset of EAE and enhance T cell proliferation and migration in EAE to aggravate the progression of the disease [31, 32]. And studies have shown that CCL-2 antibodies effectively reduce the severity of EAE [33]. CCL-3 and CCL-5 are also effective chemoattractant for Th1 cells [34]. Similarly, we concluded that treatments with anti-CCL-3 and anti-CCL-5 is effective in relieving inflammation. Interestingly, the mRNA expression of IFN chemokines (CXCL-1 and CXCL-2), and IL-17 chemokines (CXCL-9 and CXCL-10) had also significantly reduced in EAE mice after RMF exposure. We hypothesized that RMF treatment reduced chemokines of IFN and IL-17, and it was likely to have an effect impact on IFN level of Th1 cells and IL-17 level of Th17 cell population.

Meanwhile, the inhibition of peripheral T cell initiation has been identified as another effective treatment strategy for the initial stage of EAE [35, 36]. MS is related to Treg dysfunction and the enhancement of Th1 and Th17 responses, leading to the destruction of myelin, which results in neuronal injury and neuroinflammation [4], thereby promoting the progression of MS. Although the substantial difference in the amount of circulating Tregs relative to healthy controls has not been reported often in MS patients, it has been reported that these patients have a lower inhibitory capacity [37, 38]. Tregs inhibit peripheral immune responses primarily by inhibiting T helper (Th) cells [10]. This suggests that defects in Treg cells may advance the pathogenesis of MS. Mechanically, lower Treg inhibition may result in increased generation of pro-inflammatory cytokines, such as IL-17 and IFN-γ [21]. Therefore, we speculated that the inflammation caused in EAE could be inhibited by the augmentation of the number of Treg cells. We detected RMF treatment effects using flow cytometry and observed the augmentation in the number of Foxp3 cells in the spleen and in the draining lymph nodes of EAE mice. Along with a substantial rise in the manifestation levels of IL-10, TGF-β and Foxp3 mRNA, we speculated that RMF exposure could stimulate the differentiation of Treg cells in the spleen and increase its inhibitory capacity. We further verified that compared with untreated EAE mice, the proportion of IFN-γ and IL-17A expressing cells in EAE mice had significantly decreased, while that the manifestation levels of Rorc and Tbet mRNA had significantly increased after RMF exposure. It can be concluded that RMF treatment may prevent the differentiation of Th1/Th17 cell subsets. Treg cells were found to have inhibited Th cell proliferation and cytokine production via cell-cell contact mechanisms or by the generation of immunosuppressive cytokines, such as IL-10 and TGF-β [39]. It was concluded that RMF treatment may prevent the differentiation of Th1/Th17 cell subsets by promoting the differentiation of Treg cells and promoting the generation of immunosuppressive factors. On the other hand, as expected, we found using immunofluorescence that RMF exposure could cause a substantial decrease in IFN-γ and IL-17A protein levels in the spinal cord at peak EAE development. Coherent with peripheral lymphoid results, these results are consistent with our inferences.

In conclusion, our study showed that RMF treatment alleviated inflammation by promoting the demarcation of Treg cells to inhibit the generation of Th1 and Th17 cells. And RMF prevented CD4^+^ cell infiltration in the spinal cord of EAE mice by inhibiting the migration of lymphocytes with reduced chemokines. As a mild physical treatment, RMF exhibits therapeutic potential and provides an alternative form of medication for MS.

## MATERIALS AND METHODS

### Mice

Female C57BL/6 mice (6-8-week-old) were bought from the Medical Experimental Animal Center of Guangdong (Guangdong, China) [SPF, SCXK(G)2018-0002]. The recommendations of the Ministry of Science and Technology of China plus the associated ethical policies of Shenzhen University were followed for experimentations.

### EAE induction and treatment

The mice were subcutaneously injected with 200 μg MOG_35–55_ mixed in complete Freund's adjuvant comprising 4 mg/mL *Mycobaterium tuberculosis*. Pertussis toxin (500 ng /animal) was given intraperitoneally on the day of vaccination (day 0) and 48 h later. The mice were inspected everyday and graded for disease severity as mentioned below: 0 = no symptom; 1 = tail weakness; 2 = paraparesis (incomplete paralysis of one or two hind limbs); 3 = paraplegia (complete paralysis of one or two hind limbs); 4 = paraplegia with forelimb weakness or paralysis; and 5 = moribund or dead.

### RMF exposure

**Figure 9:**
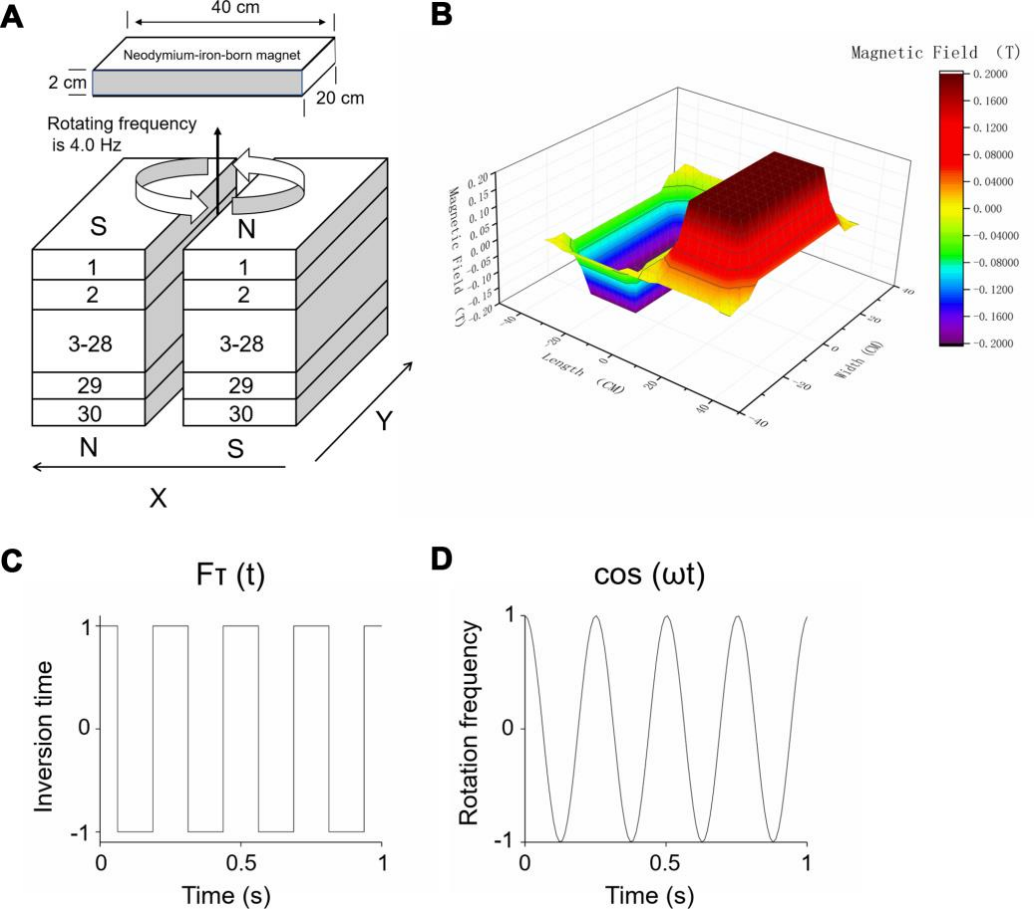
**RMF exposure device.** (**A**) Treatment tables were positioned at the central top of the device. Rotating frequency was 4.0 Hz and intensity was 0.2 T. (**B**) The magnetic field of different amplitude consisting of two overlapping components was formed above the device: (**C**) translational (with fluctuating inversion time) and (**D**) rotational (with fluctuating rotational frequencies).

The detailed structure of the RMF exposure device used in the experiments has been described previously [17, 19]. As shown in Figure 9A, the RMF was derived from two anti-parallel stacks neodymium-iron-boron permanent magnets. The mice were positioned on the treatment table for magnetic field processing [19, 21]. The intensity of RMF (Figure 9B) was acquired by means of a Hall Magnetometer (ETM-13-Achsen, Switzerland) plus a three-axis fluxgate magnetometer (GMW Associates, USA). The RMF of different amplitude consisting of two overlapping components was formed above the device: translational (with fluctuating inversion time) (Figure 9C) and rotational (with fluctuating rotational frequencies) (Figure 9D).

### Study design

The mice were randomly divided into the sham, RMF exposure, EAE and EAE+RMF exposure groups (n = 6 each), and placed in separate exposure cages. As outlined in Figure 1, the RMF and EAE and EAE+RMF group were exposed to RMF (4 Hz, 0.2 T) for 2 h daily after immunization. The sham and EAE group mice were placed in a geomagnetic field for the same schedule. Until the peak of the disease, the mice were sacrificed and related tests were performed.

### Locomoter behavioral test

On the 20^th^ day after immunization, mice were placed in Pheno Typer 3000 cages (Noduls, Netherlands) individually. After the mice had got acclimatized environment, the movement of the mice in the cage was recorder. Then, using the Ethovision XT V14 processing system (Noduls, Netherlands), a mouse behavior trajectory map was drawn, and the total distance of movement and the average of moving speed are calculated.

### Histopathology and Immunofluorescence

Cervical dislocation was performed to sacrifice the animals. The lumbar enlargement of the spinal cords were isolated from animals and instantly fixed in 4% paraformaldehyde for a minimum of 48 h. The spinal cords were frozen and cut into 6 μm sheets, stained with hematoxylin and eosin (H&E) as well as luxol fast blue (LFB). The sections were blocked using 5% goat serum for 30 min. Next, overnight incubations at 4 °C were done with anti-CD4 (CST, USA), anti-IFN-γ (Abcam, UK), anti-IL-2 (Abcam, UK), anti-IL-4 (Abcam, UK) and anti-IL-17A (Abcam, UK). Next, they were incubated with Alexa Fluor 488 secondary antibody (Abcam, UK). A fluorescence light microscope (Zeiss, Germany) was used for examination.

### T-cell proliferation

T cell propagation was detected with BrdU cell proliferation ELISA kit (colorimetric) (Abcam, UK). Lymph node-derived T cells acquired from EAE mice were re-activated with MOG35–55. After 48 h, BrdU reagent was mixed, and the cells were cultured for another 24 h. BrdU integration was measured as per supplier’s instructions.

### Cytokine examination by CBA as well as ELISA assay

Serum levels of IL-2, IL-4, IL-6, IFN-γ, TNFα, IL-17A as well as IL-10 were acquired by means of Cytometric Bead Array (CBA) mouse Th1/Th2/Th17 cytokine kit (BD, USA). IFN-γ, IL-2, IL-4 as well as IL-17A were determined by a precise ELISA kit (Invitrogen, USA) as per supplier’s instructions.

### Flow cytometry (FC)

For surface-marker staining, incubations were done with fluorochrome-conjugated antibody (BD Biosciences, USA) to APC anti-CD4, FITC anti-CD8 for 30 min on ice. For intracellular staining after fixed rupture, cells were incubated with fluorochrome-conjugated antibody (BD Biosciences, USA) to PE anti-Foxp3, PE anti- IFN-γ, PE anti-IL 2, PE anti-IL 17A for 40 min on ice. Data were obtained on a FACSAria flow cytometer (BD, USA).

### Quantitative real-time PCR

Total RNA was extricated from the spleen cells and spinal cord with TRIZOL (Sigma-Aldrich, USA), and reverse transcribed to cDNA. Quantitative PCR was accomplished utilizing Sybr Green qPCR Master Mix (DBI, Ludwigshafen, Germany). The conditions for amplification were 95°C for 3 min, 40 cycles at 95°C for 5 s, 60°C for 30 s. β-actin served as a control. All primers were manufactured by BGI (Wuhan, China) and the sequences are shown below:

**Table:** 

	**Forward primer**	**Reverse primer**
β-actin	GAGACCTCAACACCCCAG	CATCACAATGCCTGTGGTAC
T-bet	CCATTCCTGTCCTTCACCGT	CCTGTAATGCTTGTGGGCT
Foxp3	GTCTGGAATGGGTGTCCAGG	AGCGTGGGAAGGTGCAGAG
Rorc	AGCTGCGACTGGAGGACCTT	CCCGTGAAAAGAGGTTGGTG
TGF-β	AGGACCTGGGTTGGAAGTGG	AGTTGGCATGGTAGCCCTTG
IL-10	GCTCTTACTGACTGGCATGAG	CGCAGCTCTAGGAGCATGTG
CCL-2	CCCAATGAGTAGGCTGGAGA	AAGGCATCACAGTCCGAGTC
CCL-3	CAATTCATCGTTGACTATT	CAGTGATGTATTCTTGGA
CCL-5	GAGGATTCCTGCAGAGGATCAAGACAG	TCCAAAGAGTTGATGTACTCCCGAACC
CXCL-1	CTTGCCTTGACCCTGAAGCTC	AGCAGTCTGTCTTCTTTCTCCGT
CXCL-2	CCCCCTGGTTCAGAAAATCA	GCTCCTCCTTTCCAGGTCAGT
CXCL-9	TGCACGATGCTCCTGCA	AGGTCTTTGAGGGATTTGTAGTGG
CXCL-10	TGATTTGCTGCCTTATCTTTCTGA	CAGCCTCTGTGTGGTCCATCCTTG
IL-17A	TGTCTCTGATGCTGTTGCT	GTTGACCTTCACATTCTGG
IFN-γ	AGCAACAACATAAGCGTCATT	CCTCAAACTTGGCAATACTCA

### RNA-seq

Total RNA was extricated from the spinal cord with TRIZOL (Sigma-Aldrich, USA). Subsequently, RNAseq was completed by BGI (Wuhan, China).

### Statistical analysis

Statistical significance between groups were measured using two-tailed Mann-Whitney's t-test for clinical score assessment. In other experiments, One-way ANOVA followed by Dunnett’s t-test was implemented. Data is documented as the percentage or mean ± SEM. Statistical significance was considered with following values: *P < 0.05, **P < 0.01, ***P <0.001.
